# The Effect of Bulky Substituents on Two π-Conjugated Mesogenic Fluorophores. Their Organic Polymers and Zinc-Bridged Luminescent Networks

**DOI:** 10.3390/polym11091379

**Published:** 2019-08-22

**Authors:** Rosita Diana, Barbara Panunzi, Simona Concilio, Francesco Marrafino, Rafi Shikler, Tonino Caruso, Ugo Caruso

**Affiliations:** 1 Department of Agriculture, University of Napoli Federico II, 80055 Portici NA, Italy; 2 Department of Industrial Engineering, University of Salerno, 84084 Fisciano SA, Italy; 3 Department of Pharmacy, University of Salerno, 84084 Fisciano SA, Italy; 4 Department of Electrical and Computer Engineering, Ben-Gurion University of the Negev, POB 653, 84105 Beer-Sheva, Israel; 5 Department of Chemistry and Biology, University of Salerno, 84084 Fisciano SA, Italy; 6 Department of Chemical Sciences, University of Napoli Federico II, 80126 Napoli, Italy

**Keywords:** luminescent polymer, salen dyes, polymer network

## Abstract

From a dicyano-phenylenevinylene (PV) and an azobenzene (AB) skeleton, two new symmetrical salen dyes were obtained. Terminal bulky substituents able to reduce intermolecular interactions and flexible tails to guarantee solubility were added to the fluorogenic cores. Photochemical performances were investigated on the small molecules in solution, as neat crystals and as dopants in polymeric matrixes. High fluorescence quantum yield in the orange-red region was observed for the brightest emissive films (88% yield). The spectra of absorption and fluorescence were predicted by Density Functional Theory (DFT) calculations. The predicted energy levels of the frontier orbitals are in good agreement with voltammetry and molecular spectroscopy measures. Employing the two dyes as dopants of a nematic polymer led to remarkable orange or yellow luminescence, which dramatically decreases in on-off switch mode after liquid crystal (LC) order was lost. The fluorogenic cores were also embedded in organic polymers and self-assembly zinc coordination networks to transfer the emission properties to a macro-system. The final polymers emit from red to yellow both in solution and in the solid state and their photoluminescence (PL) performance are, in some cases, enhanced when compared to the fluorogenic cores.

## 1. Introduction

The development of solid state fluorescent materials, based on an organic scaffold or metal containing, is an area of research in enormous development due to the numerous applications for the study of biological [1,2,3] and photonic systems [4,5,6,7,8,9,10]. Extended π-system with donor-acceptor framework featuring a large dipole moment in the excited state are often reported as organic emitters in the solid state and as candidates for optoelectronic applications [11,12]. In particular, efficient deep red near IR photoluminescent (DR/NIR PL) emitters are chemically and thermally stable and are easily processable; thus, they are optimal candidates for fluorescence bioimaging and assay applications [13]. Among them, Schiff bases with *N*,*N*′-bis(salicylidene)ethylenediamine (salen) groups and their metal complexes have a wide variety of applications in the fields of biology [14], catalysis [15,16], materials [17], inorganic [2] and analytical chemistry [4].

To date, the challenge to obtain solid-state photoluminescent materials translates in the easy and cost-effective assembly of macro-architectures starting from small PL active molecules [18,19,20,21]. This means not only dye-doped matrixes but also covalently bonded emissive materials. 

In two of our previous works [22,23], we reported of two simply liquid crystalline dyes based on a di-cyano phenylenevinylene and an azobenzene skeleton. In both cases, the neat chromophores in the solid state were weakly luminescent, undergoing an ACQ (aggregation-caused quenching) effect, limiting the applications in devices to the diluted doped systems. 

The present paper aims to improve the PL performances of the phenylenevinylene (PV) and azobenzene (AB) emissive cores, relieving the ACQ effect, through modifications of the terminal groups of the dyes. Molecular interaction plays a key role in the properties of materials. One of the most common strategies to increase emission intensity in concentrated solution or solid state is to restrict the molecular motions (bond rotation and vibration), to prevent the nonradiative decay channels [24,25,26,27,28,29,30,31]. In some previous works, we investigated the effect of a bulky substituent on emissive dyes [32,33,34,35,36,37,38]. This structural modification reducing detrimental intermolecular interactions lead to a relevant improvement of the photophysical performances in the solid state [39]. It is well known that the addition of bulky substituent can improve PL quantum yield (PLQY) by increasing the distance between the emitting species in the solid state [40], preventing the formation of stacking interactions, and slowing down the rate of radiationless decay. More recently, another intriguing approach to enhance the PLQY of the solid compound relies on the excited-state intramolecular proton transfer (ESIPT) process [41]. The fast proton transfer in ESIPT molecules causes nonradiative deactivation, thus, most of the ESIPT-based dyes exhibit strong emission in solution but weak emission in the solid state. Introducing steric crowding and/or strong hydrogen bonding in the ESIPT dye restrict intramolecular rotation and provide good PL performance also in the solid state. 

**Scheme 1:**
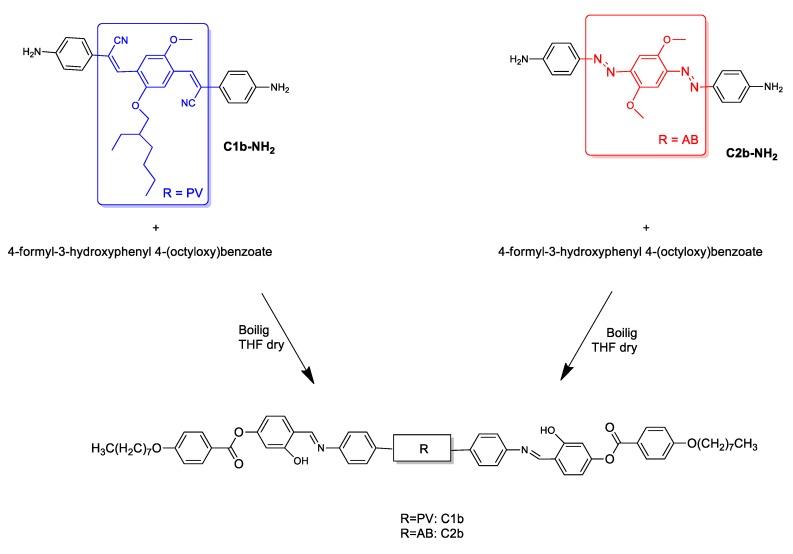
Synthetic route to the target compounds C1b and C2b.

As part of our continuous research in the field of PL materials, here, we present the Schiff base products of a substituted 2-hydroxy-benzaldehyde with diamino PV and AB based derivatives. The new fluorophores, C1b and C2b (in Scheme 1), have symmetrical bulky salen groups, potentially undergoing the ESIPT process, and flexible terminal chains. The dyes were characterized for their PL response both in solution and in the solid state. As neat solids, their PLQYs were measured and compared with the values recorded on the doped samples obtained by dispersing the dyes in conductive, non-conductive and liquid crystal (LC) polymers. 

**Scheme 2:**
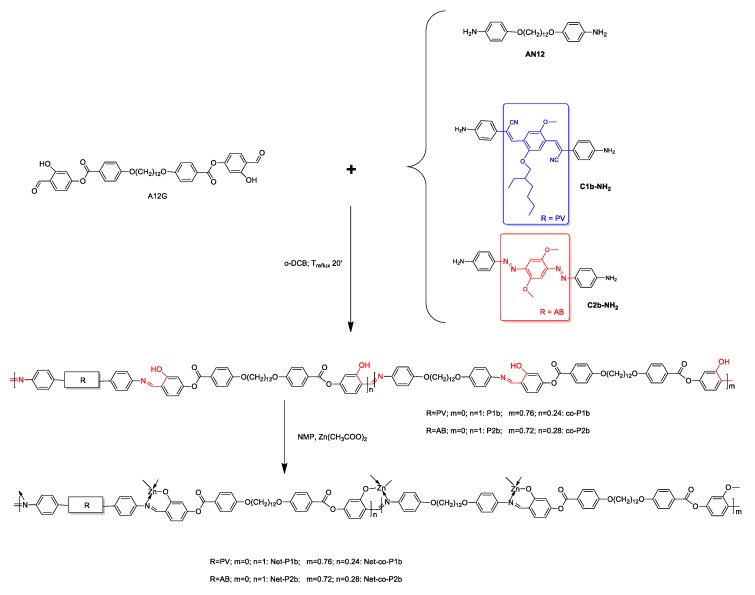
Synthetic route to the organic polymers (the chelating sites evidenced in red color) and to the networks.

Transferring and retaining/enhancing the solid-state PL performance of the microenvironment to the macro-system was the purpose for which we acquired and systematically compared the experimental data. In this perspective, PV and AB cores were covalently embedded in organic homo- and copolymers (see Scheme 2) reproducing the same emissive low molecular weight fluorophores C1b and C2b in the polymeric main-chain. These materials were prepared through polycondensation of the diamino-functionalized fluorogenic cores, and eventually a non-emissive co-monomer, and are from red to yellow emitters in solution and as solid films. From the organic polymers, zinc-driven self-assembly reaction leads to metallated networks. Zinc (II) ion linking two N,O mononegative sites of the terminal salen groups acts a cross-linker agent, producing new materials with interesting rheological properties. In this case, in situ synthesis was experimented as a novel facile route for constructing cross-linked network luminescent films as required in PL devices. The photoluminescence experiments demonstrate that the in situ deposed films are from red to yellow luminescent layers with medium PLQYs, physically and chemically stable after six months.

## 2. Materials and Methods

### 2.1. Materials

The compounds A12G, AN12 4-(octyloxy)benzoate-4-(6-hydroxy)benzaldehyde [42] and C1-NH_2_ [22] and C2-NH_2_ [23] were obtained as described in previous works. QYPDLC-102 nematic LC polymer was purchased from Canaanchem. Polymeric Matrixes: PS (molecular weight: 18,700 Da, Qingdao, China) and poly(9-vinylcarbazole) (PVK) (molecular weight: 1100 Da) were commercially available supplied by Sigma Aldrich (St. Louis, MO, USA).

### 2.2. Characterization 

A Zeiss Axioscop polarizing microscope, implemented with an FP90 Mettler heating stage, was used for optical observations. A Perkin Elmer Pyris 1 (DSC scanning calorimeter, PerkinElmer, Inc., Waltham, MA, USA) at a scanning rate of 10 °C/min, under nitrogen flow, was used to measure enthalpies and phase transition temperatures. The point at 5 wt % weight loss is assumed to represent the decomposition temperature (T_d_). Decomposition temperatures (T_d_) were defined with a Perkin Elmer TGA 4000 (thermogravimetric analysis, PerkinElmer, Inc., Waltham, MA, USA), under nitrogen flow. A Bruker Avance II 400 MHz spectrometer was used to register ^1^H NMR spectra. A Jasco F-530 spectrometer (scan rate 200 nm min^−1^, JASCO Inc., Mary’s Court, Easton, MD 21601, USA) was used to register UV-Visible spectra; fluorescence spectra were registered with a spectrofluorometer Jasco FP-750 (JASCO Inc., Mary’s Court, Easton, MD 21601, USA) (excitation wavelengths set at the samples’ absorption maxima, scan rate 125 nm min^−1^). An Ubbelohde viscometer was used to determine the inherent viscosity (η_inh_), at 40 °C in *N*-methyl pyrrolidinone (NMP), of the organic polymers P1b, P2b, co-P1b and co-P2b. Vapor pressure osmometry at 7000 °C in Dimethyl sulfoxide (DMSO) was employed to measure molecular weights by a Knauer apparatus.

### 2.3. Thin Films for Optical Measurements and PLQY Setup

All thin film samples used for PLQY measurements were prepared by an SCS P6700 spin-coater apparatus. By the spin-coating technique operating in two steps (600 rpm for 60 s and 2000 rpm for 60 s), they were deposed on quartz slides C1b and C2b as neat samples dissolved in NMP and dissolved at 10% by weight in PS or PVK. The samples were annealed for 10 min at 100 °C. Thin films were obtained by spin-coating of a 10mg/mL solution of the organic polymers in NMP operating in two steps (600 rpm for 60 s and 2000 rpm for 60 s); the samples were annealed 150 °C for 5 min. The in-situ reaction of zinc-driven self-assembly of the polymers afforded the networks by spin-coating the reaction mixture (see Section 2.8) onto quartz slides operating at 2000 rpm for 60 s and annealing of the films at 150 °C for 10 min. The films were washed with isopropyl alcohol twice and dried at 60 °C for 20 min in vacuum. Doped LC samples were obtained by dissolving C1b or C2b (1% by weight) and commercially available nematic LC polymer, using Tetrahydrofuran (THF) as a solvent, and removing the solvent at 100 °C. The mixture was deposed between quartz slides for the measurements.

A setup similar to the one proposed by de Mello et al. [43] was used to run PL efficiency measurements. The system is composed of an exciting laser (at 405 nm or 475 nm) and a spectrophotometer (BLACK Comet Stellarnet Inc, Tampa, FL, USA); it collects the direct photoluminescence and the scattering effect of the integrating sphere photoluminescence (part of the system, Stellarnet Inc., Tampa, FL, USA). Five different points on the sample were considered to measure the emission.

### 2.4. Cyclic Voltammetry

All electrochemical measurements were performed with 663VA stand interfaced to Autolab PGSTAT302N potentiostat-galvanostat (Metrohm) controlled by NOVA software. The cell consisted of three electrodes. A platinum bar was used as the auxiliary electrode and a platinum wire as the quasi-reference electrode calibrated against the redox potential of ferrocenium/ferrocene redox couple, Fc^+^/Fc [44]. The working electrodes were platinum disk electrodes (2 mm, purchase from Metrohm AG, 9100 Herisau, Switzerland), coated by a polymer film. The working electrodes were prepared by pipetting 10 μL of polymer solution NMP, prepared at 80 °C by solving 5.00 mg of polymer in 500 µL of NMP. After the drying period (15 h at 80 °C), the working electrode was immersed in an electrolyte solution, 0.1 M tetrabutylammonium hexafluorophosphate in acetonitrile. The electrolyte solution was purged with argon for 15 min before the measurements to remove the oxygen.

### 2.5. Theoretical Calculations

Quantum-mechanical calculations were carried out at theory level DFT/B3LYP; Jaguar package, Schrödinger Release 2017-4 [45] was used. B3LYP functional and LACVP** basis set were used to optimize molecular geometry. The NBO approach was applied to perform charges assignment. Single-point calculations were performed on optimized geometries to re-evaluate the energies; Dunning's correlation-consistent triple-ζ basis set cc-pVTZ (-f), which includes a double set of polarization functions, was used. Proper convergence to local minima was confirmed by calculating vibrational frequency, based on analytical second derivatives at the B3LYP/6-31G**(LACVP**) level of theory. Zero-point energy (ZPE) and entropy corrections at 25 °C were also derived using vibrational frequency calculations, using non-scaled frequencies. Vertical excitation energies, computed at neutral compound geometry with TD-DFT and Tamm-Dancoff [46] approximation, were used to obtain absorption values. Poisson Boltzmann Solver (PBF) [47] was used to model the solvent ethanol. Computed redox data was used to calculate “scaled” HOMO and LUMO energies, via the expressions:

Absolute_Electrode = NHE_Energy + Electrode_Potential

Orbital_Energy = Absolute_Electrode + Redox_Potential

where NHE_Energy is the energy of the NHE (Normal hydrogen electrode) electrode in water (−4.28 V), Electrode_Potential is the potential of the chosen electrode relative to NHE.

### 2.6. Synthesis of C1b and C2b

A similar procedure was adopted for the synthesis of both dyes, by reacting the diamino derivative (respectively, C1-NH_2_ and C2-NH_2_ in Scheme 1) with 4-(octyloxy)benzoate-4-(6-hydroxy) benzaldehyde in 5% stoichiometric excess. As an example, the synthesis of C1b is reported. 500 mg of the diamino derivative (0.96 mmol) and 711 mg (1.92 mmol) of 4-(octyloxy)benzoate-4-(6-hydroxy)benzaldehyde were dissolved in THF dry (10.0 mL). After 30 min at reflux under stirring, the mixture was cooled to room temperature leading to the formation of a red crystalline solid. The compound was recovered by filtration and recrystallized by THF. Yield: 80%; mp: 221 °C (⊗H = 30.90 J/g); T_d_ = 311 °C. ^1^H NMR (400 MHz, DMSO-d_6_, 25 °C, ppm): 0.88 (m, 12H), 1.30 (m, 24H), 1.44 (m, 4H), 1.76 (m, 5H), 3.95 (m, 5H), 4.00 (t, 4H), 6.79 (s, 2H), 7.00 (t, 4H), 7.11 (m, 4H), 7.20 (m, 4H), 7.50 (d, 4H), 7.70 (d, 2H), 8.13 (dd, 6H), 9.07 (s, 2H), 13.60 (s, 2H). Elemental analysis calculated (%) for C_77_H_84_N_4_O_10_: C, 75.46; H, 6.91; N, 4.57; found: C, 75.78; H, 6.66; N, 4.20.

C2b: Yield: 78%; mp: 181 °C (⊗H = 71.90 J/g); T_d_ = 301 °C. ^1^H NMR (400 MHz, DMSO-d_6_, 25 °C, ppm): 0.87 (m, 6H), 1.28 (m, 16H), 1.43 (m, 4H), 1.74 (m, 4H), 3.90 (m, 6H), 4.10 (t, 4H), 6.58 (s, 2H), 6.91 (t, 4H), 7.11 (m, 4H), 7.28 (m, 4H), 7.48 (m, 4H), 7.54 (dd, 2H), 8.07 (t, 4H), 9.07 (s, 2H), 13.60 (s, 2H). Elemental analysis calculated (%) for C_64_H_68_N_6_O_10_: C, 71.09; H, 6.34; N, 7.77; found: C, 71.01; H, 6.16; N, 7.20.

### 2.7. Synthesis of Organic Polymers

Scheme 2 shows the basic polymerization procedure for all the polymers; the following is a detailed description for polymer P1b. A total of 0.464 g (0.68 mmol) of dialdehyde A12G was dissolved in 5 mL of boiling o-dichlorobenzene, and 0.354 g (0.68 mmol) of diamine C1-NH2 added under a nitrogen atmosphere. The solution was kept at boiling temperature for 20 min to carry out polymerization and was then poured into n-hexane. The precipitated polymer was washed twice in n-hexane and oven-dried at 100 °C. The same procedure was used for preparing the copolymers, utilizing 10% by weight of C1b-NH_2_ or C2b-NH_2_, diamine AN12, and the stoichiometric amount of dialdehyde A12G. The description is reported for polymer co-P1b. To 0.302 g (0.58 mmol) of diamine C1-NH_2_ and 0.842 g (2.19 mmol) of AN12 dissolved in 10 mL of o-dichlorobenzene, 1.891 g (2.77 mmol) of A12G was added under a nitrogen atmosphere. The polymerization was carried out for 20 min at boiling temperature and the solution is then poured into n-hexane. The precipitated polymer was washed twice in n-hexane and oven-dried at 100 °C. Yields are virtually quantitative in all cases. ^1^H NMR (400 MHz, TCE-d_2_, 25 °C) spectra of the copolymers are reported in Figure S1 of Supplementary Materials. Co-P1b: T_m_ = 126 °C; T_d_ = 310 °C. Co-P2b: T_m_ = 164 °C; T_d_ = 324 °C.

P1b: T_m_ = 186 °C; T_d_ = 345 °C (calculated as the 5% weight loss temperature in N_2_). ^1^H NMR (400 MHz, TCE-d_2_, 25 °C, ppm): 0.88 (m, 6H), 1.30 (m, 20H), 1.44 (m, 4H), 1.76 (m, 5H), 3.83 (m, 5H), 4.00 (m, 4H), 6.67 (s, 2H), 6.82 (m, 4H), 7.18 (m, 8H), 7.50 (d, 4H), 8.03 (m, 8H), 9.84 (s, 2H), 11.60 (s, 2H). P2b: T_m_ = 232 °C; T_d_ = 333 °C (calculated as the 5% weight loss temperature in N_2_). ^1^H NMR (400 MHz, TCE-d_2_, 25 °C, ppm): 1.28 (m, 14H), 1.44 (m, 2H), 1.79 (m, 4H), 3.93 (m, 6H), 4.02 (t, 4H), 6.60 (m, 4H), 6.85 (d, 4H), 6.97 (s, 2H), 7.13 (s, 2H), 7.29 (d, 2H), 7.62 (d, 2H), 8.09 (m, 6H), 8.50 (s, 2H), 9.84 (s, 2H), 11.21 (s, 2H).

### 2.8. Synthesis of the Networks

Cross-linking of the polymers was performed in solution following the same basic procedure for all the polymers. A detailed description is reported for Net-P1b. To a solution of 0.120 g (0.103 mmol) of P1b dissolved in 10 mL of hot (150 °C), NMP 0.019 g (0.103 mmol) of zinc (II) acetate dissolved in 1 ml of NMP. After 5 min under vigorous stirring, the solution was poured into methanol and the precipitated network washed twice in methanol and oven-dried at 150 °C. Yields are virtually quantitative. Zn % as ZnO was evaluated by TGA analysis of the samples heated up to 900 °C. Net-P1b: calculated 6.59%; found 6.22%. Net-P2b: Zn%: calculated 7.47%; found 7.40%. Net-co-P1b: calculated 7.31% found 7.20%. Net-co-P2b: calculated 7.42% found 7.31%. Decomposition temperature for all networks is above 350 °C.

## 3. Results and Discussion

### 3.1. Synthesis and PL Properties of C1b and C2b

To assemble efficient and stable emissive layers, efforts must devote to the design and preparation of the active luminescent units. We explored the potential of PV [22,32] and AB [23,48] fluorogenic cores embedded in the low molecular weight molecules C1b and C2b, reported in Scheme 1. Both dyes are the symmetrical Schiff base products of a substituted 2-hydroxy-benzaldehyde with the diamino derivatives C1-NH_2_ and C2-NH_2_ (Scheme 1). The benzoyloxy groups and the alkoxy substituents on the central ring were added to restrict rotation/isomerization and guarantee fluorescence. Terminal flexible chains were introduced to increase solubility and to promote the LC phase.

The identification and evaluation of the purity degree were assessed by ^1^H-NMR and elemental analysis. By optical observation and DSC methods, the expected liquid-crystalline phase behavior was revealed in both cases, with a large stability range. Compound C1b melts to a marble nematic texture at 221 °C and C2b at 181 °C under polarized light (Figure S2 of Supplementary Materials). Decomposition occurs before isotropization, above 300 °C for both monomers. In both cases, the dyes result emissive in the nematic phase.

Photophysical measurements were performed in the solution and in the solid state. As shown in Table 1, emission maxima of C1b are always red-shifted respect to C2b, and the same general behavior has been found in the polymeric materials (see Section 3.2).

**Figure 1:**
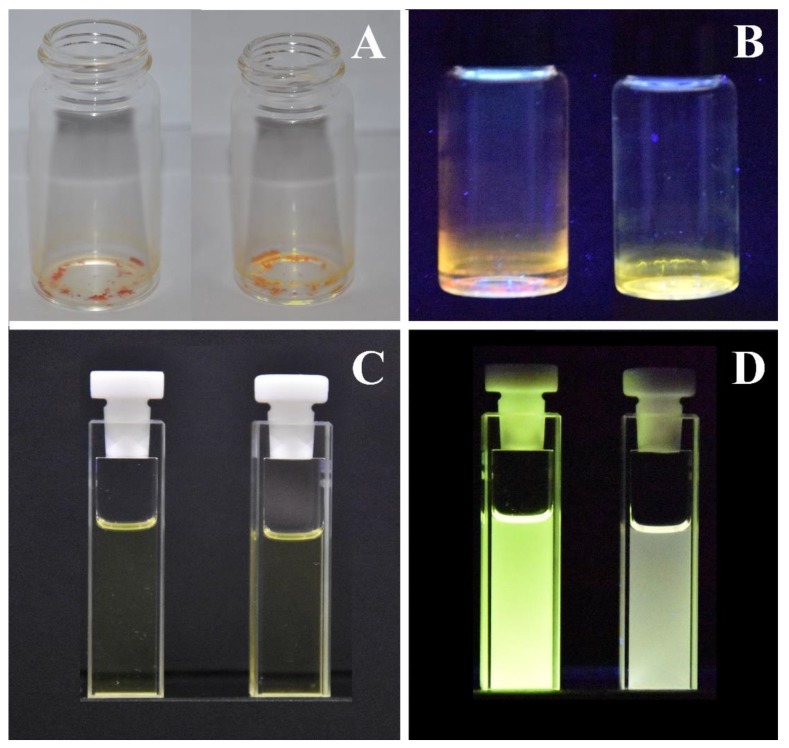
Crystalline samples of C1b (**A** on the left) and C2b (**A** on the right) in visible light and under 365 nm UV lamp light (**B**). The same samples dissolved in a dilute chloroform solution in natural (**C**) and under 365 nm UV lamp light (**D**).

In the solution, C1b and C2b are weak yellow emitters (Figure 1), with a poor solvatochromic effect observed, depending on the polarity of the solvent (red-shift for absorption maxima and blue-shift for emission maxima). Stoke’s Shifts from 81 nm to 164 nm have been calculated respect to absorption maxima in the different solvents. PL quantum yields of the dyes are, respectively, 5% and 3%. They were measured in a chloroform solution by relative methods using as standard quinine sulfate [49].

The photophysical characterization of the crystalline samples was performed on spin-coated thin films of the neat solids. With respect to analogous PV and AB based dyes without bulky groups [23], the benzoyloxy moiety introduces steric and therefore, electronic changes. As discussed below, the whole aromatic skeleton of the molecules constitutes a conjugated backbone where HOMO is delocalized. The new push-pull system leads to an improvement in PL emission intensity and a blue-shift of the emission maxima respect to the non-bulky dyes [22,23]. Respectively, red and orange-yellow bright luminescence (see Figure 1) was recorded on crystalline samples of neat C1b and C2b with 25% and 22% PLQYs, PL performance resulting about six times higher respect to the non-bulky dyes. The remarkable Stokes Shifts recorded on the neat samples (138 nm and 208 nm, respectively, from excitation to emission maximum wavelength) are a desirable hallmark of a PL device, able to eliminate spectral overlap between absorption and emission phenomena and to improve the intensity and color purity of the emission.

In our preliminary works, we found that luminescence can be enhanced in the doped systems where the ACQ effect decreases dramatically [22,23]. We employed the organic dyes C1b and C2b as dilute dopants (10% by weight) in polymeric matrices, in particular in non-conductive polystyrene (PS) and in hole-transporting conductive polymer poly-*N*-vinylcarbazole (PVK), typically employed to produce emissive layers in LEDs and OLEDs [50,51,52]. The doped layers are from orange yellow to yellow-green emitters with Stoke’s Shift from 70 to 93 nm (calculated from excitation to maximum emission wavelength). The best result was due to the best balance from dilution degree and interaction with host matrix. As expected, luminescence in C1b-doped polymers was enhanced in both the polymeric matrices, reaching the noteworthy value of 88% in PVK. Analogous behavior was detected for the non-bulky PV derivative. On the contrary, emission results preserved (in PVK) or slightly decreased (in PS) in 10% dispersion of C2b dye, due to different interaction between the AB dye and the host polymer [22,23]. 

To date, an increasing interest for composites based on liquid crystals (LC) polymers was evidenced [53,54,55]. The new soft matter-based devices technologies require soluble, stable, and highly efficient dyes for the LC phase. Emissive dopants in polymeric LC mixture provide luminescent liquid crystals (LLCs) highly desirable for light-emitting devices [56,57]. In the nematic phase, C1b and C2b are luminescent liquid crystals (LLCs), showing intrinsic light emission and supramolecular organization [58,59]. In the field of optoelectronic applications, the cutting-edge technology is represented by the incorporation of luminophores into liquid crystal matrix to prepare Photoluminescent Liquid Crystal Displays (PLLCDs) or for optical temperature sensing systems [60,61]. Dye solubility in the LC host matrix is one of the main limits. For this reason, the aim of LC display technology and temperature-responsive PL modulator/switch probes is a soluble fluorophore, that does not show ACQ effects at the required concentrations [62,63]. In our experiments, the two dyes were dissolved at 25 °C in a commercially available nematic polymer (QYPDLC-102, T_i_ = 113 °C) at 1% by weight even if their solubility enables to increase concentration up to 10 times. The isotropization temperatures of the doped LC polymer are substantially preserved. The homogeneous room temperature LC materials are respectively orange-yellow and yellow in natural light. In the nematic phase, they show, respectively, yellow and bright yellow-green fluorescence.

**Figure 2:**
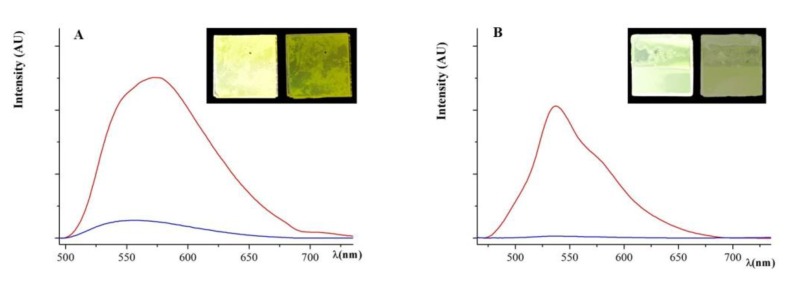
Fluorescence spectra recorded on 1% doped LC sample of C1b (**A**) and C2b (**B**) before (red curve) and after isotropization (blue curve). Inset: the same sample deposed between quartz slides under 365 nm UV light before (**left**) and after (**right**) isotropization.

Quantitative measurement was obtained by recording the fluorescence spectra on the same doped LC sample irradiated at a maximum of absorbance before and after isotropization. PLQYs recorded on the nematic films reach the good values of respectively 50% and 41% (see Table 1) with Stoke’s shifted emission of 111nm and 98 nm. PL emission maxima drastically decrease (to 10% and 1%, respectively) in on-off PL switch mode after the nematic order was lost, as can be seen with the naked eye (Figure 2). Reversibility of the process was checked by twenty cycles of on-off PL switch route.

### 3.2. Synthesis and PL Properties of Organic Polymers 

**Figure 3:**
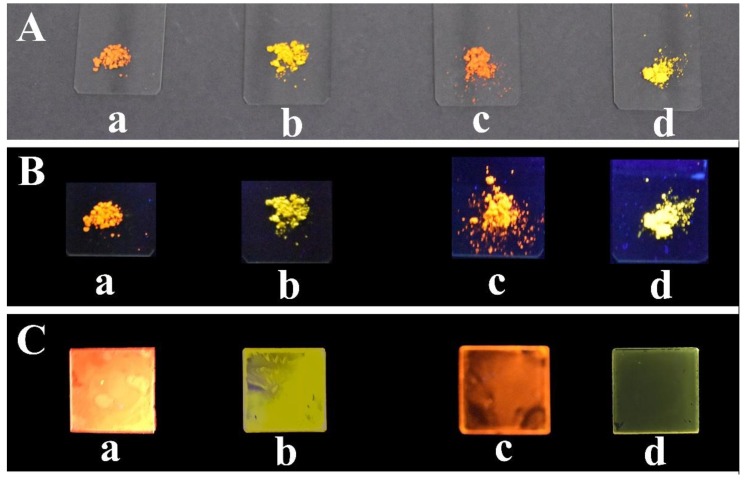
From left to right: P1b (a), P2b (b), co-P1b (c) and co-P2b (d) powder samples in natural light (**A**) and under 365 nm UV lamp (**B**). The correspondent cross-linked samples obtained by in situ deposition on quartz slides (**C**) under a 365 nm UV lamp.

The fast-growing field of organic light emitting diodes OLEDs has led to the synthesis of numerous new luminescent materials [11,12,64], in which fluorophores are covalently bonded in a polymer chain, in the attempt to transfer the PL properties of low molecular weight fluorogenic cores into a macroscopic system. We engineered and synthesized both organic homo- and copolymers based on the PV and AB emissive moieties to obtain emissive materials whose processability, stability, and reproducibility could not be guaranteed by doped materials. The synthetic route for the organic homo- and co-polymers P1b, P2b, co-P1b, and co-P2b have been summarized in Scheme 2. The four polymers are segmented main-chain organic polymers obtained by condensation of the diamino derivatives and a di-carboxaldehyde with a flexible spacer, A12G. The same fluorogenic cores with the bulky groups and ESIPT undergoing sites are repeated in all the polymers. In the copolymers, the fluorophore moieties C1-NH_2_ or C2-NH_2_ were diluted, respectively, at 24% and 28% in moles employing a di-aniline with a flexible spacer, AN12, to make the whole system more soluble. As expected, all the polymers are orange-yellow powder and display bright luminescence from red to yellow (see Figure 3).

The homopolymers show some crystallinity and an LC behavior evident respectively from 200 e 230 °C. After melting the optical observation shows a poorly mobile nematic texture. Decomposition takes place before isotropization, above 330 °C. The inherent viscosity of P1b and P2b (η_inh_) was measured at 40 °C in NMP achieving respectively 2.90 and 2.80 dL/g values, as expected for medium molecular weight. The copolymers are materials representative of the LLCs class. Their LC phase is preserved and greatly enhanced respect to the homopolymers. Polymers co-P1b and co-P2b are semi-crystalline and in optical microscopy they show, after melting, a more mobile nematic Schlieren phase stable for about 150 °C, with isotropizations barely observable, respectively, at 307 °C and 320 °C, overlapped to decomposition and not detectable in DSC analysis (as an example, see Figure S3 of Supplementary Materials). The inherent viscosity measured at 40 °C in NMP provides 2.00 and 1.50 dL/g, respectively, again in the range of medium molecular weight. Homo- and copolymers are soluble in dimethyl sulfoxide. Vapor pressure osmometry at 7000 °C of DMSO solutions was utilized to measure molecular weights. For all polymers, M, was found from 19,000 to 20,000 Da.

As for the PL properties (summarized in Table 2, for polymers and networks) in all cases, PV based materials show red-shifted emission respect to AB based polymers and networks. The homopolymers are weak yellow emitters in solution, with broad absorption bands in NMP, o-dichlorobenzene (o-DCB) or DMSO and DMF, solvents typically employed for the deposition of emissive thin films in devices. Due to their conformational flexibility, the two copolymers are notably more soluble with respect to the homopolymers. They can be also dissolved in THF, dioxane, or chloroform and display, respectively, orange and yellow emission more intense respect to P1b and P2b. Their PLQYs measured in NMP solutions are 4.5% and 3%, respectively. 

The spin-coated films obtained from NMP or o-DCB solution of homo- and copolymers observed by optical microscopy appear homogeneous up to 2 μm. PLQYs measured on thin films of P1b and P2b are, respectively, higher (34%) and quite similar (20%) to those of the monomers C1b and C2b (see Table 2). Despite the best processability, the copolymers are more diluted systems; not unexpectedly, thin films of the copolymers are less emissive and PLQYs ranges from 10% (co-P1b) to 14% (co-P2b). All the organic polymeric materials may be regarded as medium intensity red to yellow emitters.

### 3.3. Synthesis and PL Properties of the Zinc-Bridged Networks

Recently, in-situ polymerization/crosslinking approach was used as a new technique to fabricate the emitting layers for LEDs, OLEDs, and other PL devices with high performance [10,13,14,65]. The in-situ synthesis and deposition of an active network film onto the electrodes in one-step is highly attractive due to its easiness and low-cost and lead to homogeneous and very strong glassy films. 

Four supramolecular cross-linked networks were fabricated from the fluorescent polymers and zinc (II) acetate salt as cross-linker. This chemistry provides the advantage of not requiring initiators or elevated temperature so that being fully compatible with the fabrication of PL devices. By employing all the salen chelating sites (evidenced in red in Scheme 2), the organic polymers have been used as precursors to the preparation of luminescent 100% crosslinked network films based on the same PV and AB cores. Through carefully controlling the in-situ reaction conditions, the self-assembly of the polymers and zinc ion as linker has afforded the emissive glassy films of the networks depicted in Scheme 2.

In Figure 3, the red to yellow emissive cross-linked films were photographed in natural light and under a UV-vis lamp at 365 nm. We also isolated network samples by precipitation in ethanol of the reaction mixture of the organic polymers with zinc acetate for further characterization. The samples are amorphous, as supported by X-ray powder data, and showed no DSC signal up to decomposition, above 350 °C. TGA analysis provided zinc content in the materials in good agreement with the calculated values.

The role of zinc (II) ions in the construction of PL structures previously attracted our attention [66,67,68]. The use of a closed-shell d^10^ metal ion is usually a cause for increasing rigidity of the molecular structure with a decreased probability of non-radiative decay of the excited states. This phenomenon was expected to enhance the fluorescence of the ligands. On the other hand, regarding the networks, it was demonstrated that for a high concentration of cross-linker, the fluorescence intensity of polymer decreases [69]. Not unexpectedly, compared to the polymers, our thick crosslinked networks have weaker fluorescence due to the aggregation of polymer chains, which causes a strictly interacting of the PL active moieties. In conclusion, the homopolymers, containing more emissive groups respect to the copolymers, have higher PLQYs. The networks benefit from the formation of a rigid system after zinc binding, but suffer from the excessive aggregation of the chains. This is the possible explanation why all networks samples do not exceed 10% PLQYs.

Nevertheless, the better features of the crosslinked systems are their physical and chemical stability, high cohesion, and adherence to the substrate. The cross-linked films are insoluble in common organic solvents and not prone to swelling. Dimensional stability and PL response did not change after six months at 50 °C. Properly modulating of cross-linking degree could be the right balance between structural stability and PL response for actual employ in devices.

### 3.4. DFT Analysis

**Figure 4:**
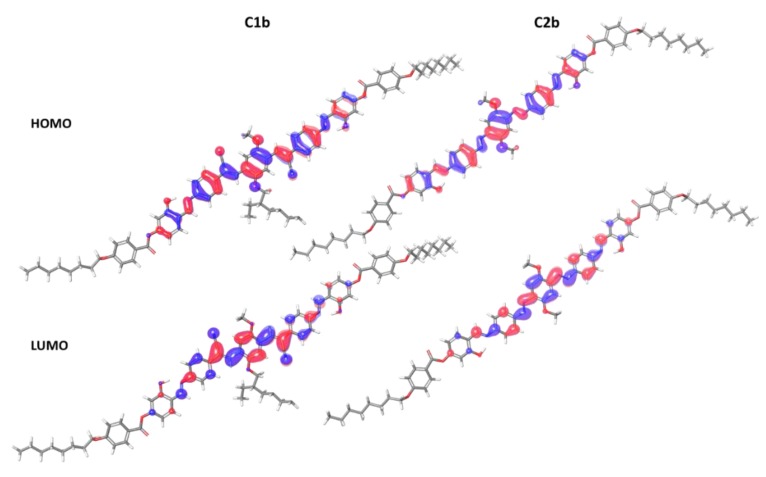
Representation of HOMO and LUMO orbitals for C1b and C2b.

C1b and C2b monomers excitation energies were calculated with the TDDFT approach at the DFT level. The adiabatic local density approximation was used; it allows to compute reliable predictions for excitation energies and oscillator strengths. The simulated solvent was ethanol. The monomers show comparable frontier orbitals (Figure 4, Table 3). HOMO delocalization is placed over the entire conjugated backbones; for C1b, cyano groups give a small contribution. LUMO is primarily delocalized over the monomers’ central sections, over C1b cyano groups (that are electron-withdrawing), and over C2b diazo groups. HOMO→LUMO is the main transition for the two monomers. The most meaningful optoelectronic properties of the monomers are shown in Table 3. Figure 4 shows the effect of the cyano group (C1b) on the electronic distribution. HOMO-LUMO gap of C1b is smaller compared to C2b; there is also a red-shift in the absorption spectrum. The computed fluorescence values are lower than the experimental values, possibly as a result of an inadequately large basis set. Thanks to DFT calculations, it is possible to study the first oxidation potential of the compound, allowing to clarify its electron-donating properties. In a redox reaction, the electron withdrawal is done from the compound’s HOMO.

### 3.5. Electrochemical Bandgaps of Organic Polymers

The conducting and electronic properties of the polymers are directly related to their bandgap energy (gap between HOMO and LUMO). Therefore, conjugated polymers showing intrinsic conductivity or low bandgap are highly required in PL devices. In this work, the energy levels were calculated as electrochemical bandgap, ΔE_EC_, [70], and optical gap, ΔE_opt_, [71] by using cyclic voltammetry, CV, and molecular spectroscopy, respectively [72]. The values are reported in Table 4.

The bandgaps obtained by cyclic voltammetry are in general lower than the optical bandgaps with a systematic deviation of *ca* − 1.4 eV. This discrepancy is entirely due to the solvation and the charge effects because in CV measurements the molecules are in contact with a conductive metal electrode and dissolved in an electrolytic solution [73]. Then, the C1b and C2b embedment in organic polymers involve a small increase of the energy gap of *ca* + 0.7 eV, the materials presenting a desirable low bandgap [74].

## 4. Conclusions

We obtained two new symmetrical salen fluorophores containing PV or AB moieties and bulky terminal substituents. PL performances of the small molecules were investigated in solution, as neat crystals and as dopants in polymeric matrixes. Red to yellow fluorescence was observed, and 88% PLQY value measured in the solid state for the brightest emissive 10% doped PVK film. As dopants of a nematic polymer, a remarkable on-off PL switch ability was detected. The presence of the cyano group has proved effective in modulating the optoelectronic properties and leaves ample scope for designing new materials with the desired properties. The PV and AB fluorogenic cores have been covalently incorporated into organic polymers and zinc crosslinked nets. This allowed the production of high PLQY polymeric materials. The obtained materials are red to yellow fluorophores in solution and the solid state, with medium PL performances, and low bandgap measured by CV. The networks, obtained by in-situ crosslinking approach, display weak fluorescence compared to the polymers although exhibit large physical and chemical stability.

## Figures and Tables

**Table 1:** Optical data of C1b and C2b in solution and as neat and dispersed samples.

Sample	λ_abs.sol_ (nm) ^a^	λ_em.sol_ (nm) ^b^	PLQY% ^c^	Solvent	λ_abs.film_ (nm) ^d^	λ_em.film_ (nm) ^e^	PLQY% ^f^	Matrix ^f^
C1b	369	533	5.00 ± 0.02	Chloroform	(403)474 ^j^	613 ^j^	25 ± 1 ^j^	neat ^j^
	373	529		THF	470 ^jj^	566 ^jj^	30 ± 1 ^jj^	PS ^jj^
	396	523		NMP	460 ^jjj^	566 ^ijjj^	88 ± 5 ^jjj^	PVK ^jjj^
	-	-		-	460 ^jv^	571 ^jv^	50 ± 5 ^jv^	PDLC ^jv^
C2b	420	534	3.00 ± 0.02	Chloroform	446 ^j^	613 ^j^	22 ± 1 ^j^	neat ^j^
	422	512		THF	429 ^jj^	566 ^jj^	10 ± 2 ^jj^	PS ^jj^
	430	511		NMP	429 ^jjj^	566 ^ijjj^	21 ± 2 ^jjj^	PVK ^jjj^
	-	-		-	414 ^jv^	571 ^jv^	41 ± 5 ^jv^	PDLC ^jv^

^a^ Wavelength of UV-Visible absorbance maxima in solution; ^b^ Wavelength of emission maxima in solution; ^c^ Photoluminescence quantum yield (PLQY) in chloroform solution measured by relative methods using as standard quinine sulfate; ^d^ Wavelength of UV-Visible absorbance maxima on thin film; ^e^ wavelength of emission maxima on thin film; ^f^ PL quantum yield on thin film; ^j^ neat sample; ^jj^ sample dispersed in PS 10% by weight; ^jjj^ sample dispersed in PVK at 10% by weight; ^jv^ sample dispersed in QYPDLC-102 nematic polymer at 1% by weight. Less intense maxima in brackets.

**Table 2:** Optical data of homo- and co-polymers and the derived networks.

Compound	λ_abs.sol_ (nm) ^a^	λ_em.sol_ (nm) ^b^	PLQY% ^c^	λ_abs.film_ (nm) ^d^	λ_em.film_ (nm) ^e^	PLQY% ^f^
P1b	321 (365,433)	532	2.00 ± 0.02	466	606	34 ± 2
P2b	326–420	498	1.30 ± 0.02	448	560	20 ± 2
co-P1b	346 (453)	518–616	4.50 ± 0.02	444	596	10 ± 1
co-P2b	348 (429)	511	3.00 ± 0.02	435	551	14 ± 2
Net-P1b	-	-	-	460	555	8.0 ± 1
Net-P2b	-	-	-	435	490	7.0 ± 1
Net-co-P1b	-	-	-	395	597	9.0 ± 1
Net-co-P2b	-	-	-	397	552	10 ± 1

^a^ Wavelength of UV-Visible absorbance maxima in NMP solution, less intense maxima in branch or range; ^b^ wavelength of emission maxima in NMP solution; ^c^ wavelength of UV-Visible absorbance maxima on thin film, less intense maxima in branch; ^d^ wavelength of emission maxima on thin film; ^e^ wavelength of emission maxima on thin film; ^f^ PL quantum yield on thin film.

**Table 3:** Electro-optical properties calculated on C1b and C2b.

Properties	C1b	C2b
Oxidation Potential (eV)	1.27	1.26
Reduction Potential (eV)	−0.92	−1.00
Hole Reorganization Energy (eV)	0.28	0.40
Electron Reorganization Energy (eV)	0.22	0.29
Triplet Energy (eV)	1.49	1.00
λ_max_ (nm)	516	509
E_max_ (nm)	565	550
Scaled HOMO (eV)	−5.55	−5.54
Scaled LUMO (eV)	−3.36	−3.28
HOMO-LUMO (eV)	2.18	2.26
Triplet Stabilization Energy (eV)	0.35	1.04
Hole Extraction Potential (eV)	5.92	5.82
Triplet Reorganization Energy (eV)	0.62	2.05
Electron Extraction Potential (eV)	−2.08	−2.05

**Table 4:** Energy bandgaps calculated using voltammetric (ΔE_EC_) and optical (ΔE_opt_) data, compared to the relative difference with the theoretical C1b and C2b HOMO-LUMO values (ΔE_opt_ − ΔE_theor_), reported in Table 3.

Polymers	ΔE_EC_ (eV)	ΔE_opt_ (eV)	ΔE_opt_ − ΔE_theor_ (eV)
P1b	1.9	2.9	0.7
P2b	1.8	3.0	0.7
coP1b	1.2	2.7	0.5
coP2b	1.4	2.9	0.6

## Supplementary Materials

The following are available online at https://www.mdpi.com/2073-4360/11/9/1379/s1, Figure S1. ^1^H NMR spectra of copolymers co-P1b and co-P2b; Figure S2. Polarized optical microscopy observation of the LC texture of C1b and C2b; Figure S3. DSC thermograms of the copolymers.
