# Supplementary material for: The Effect of Bulky Substituents on Two π-Conjugated Mesogenic Fluorophores. Their Organic Polymers and Zinc-Bridged Luminescent Networks
Source: Polymers (Basel). 2019 Aug 22;11(9):1379. doi: 10.3390/polym11091379 (PMC6780212; doi:10.3390/polym11091379)
Supplement: Supplementary file 1 [file polymers-11-01379-s001.pdf]

polymers

Supplementary Materials: The effect of Bulky Substituents on Two π-Conjugated Mesogenic Fluorophores. Their Organic Polymers and Zinc-Bridged Luminescent Networks.

Rosita Diana [1], Barbara Panunzi [1], Simona Concilio [2], Francesco Marrafino [3], Rafi Shikler [4], Tonino Caruso [5] and Ugo Caruso [6,*]

Received: 8 July 2019; Accepted: 20 August 2019; Published: date

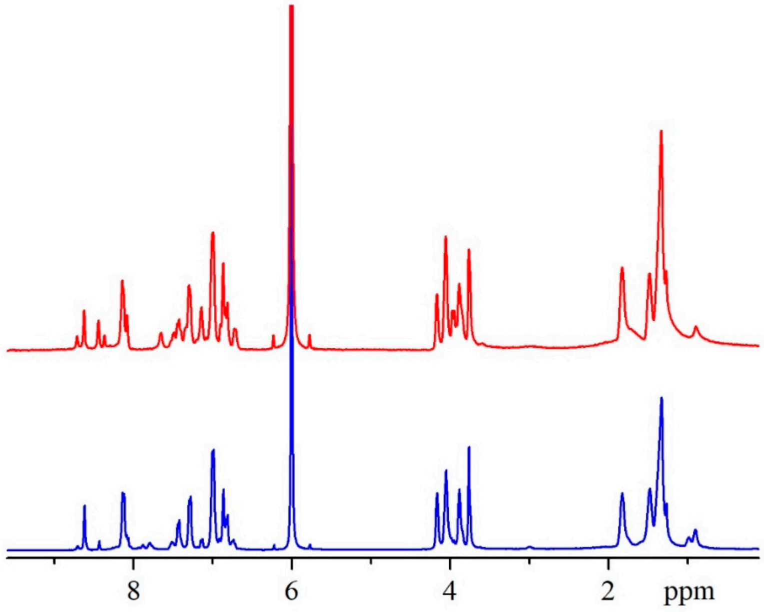

Figure S1. ^1H NMR (400 MHz, TCE-d_2, 25 °C) spectra of co-P1b (curve blu) and spectra of co-P2b (curve red).

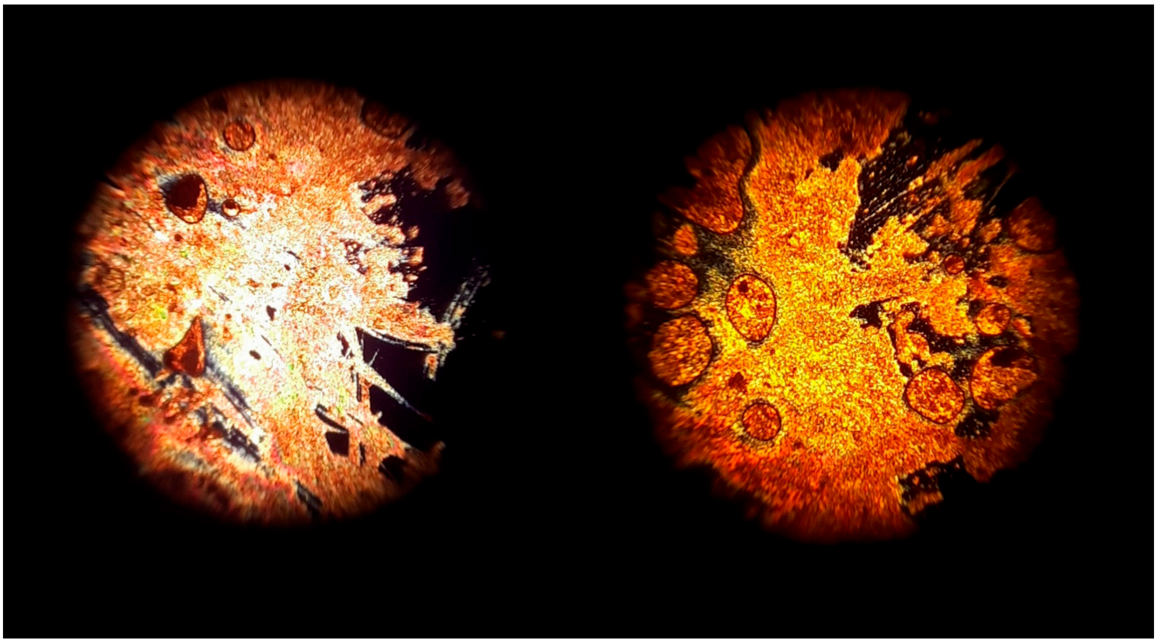

Figure S2. Marble nematic texture at 225 °C and C2b at 200 °C under polarized light of C1b (on the left) and C2b (on the right).

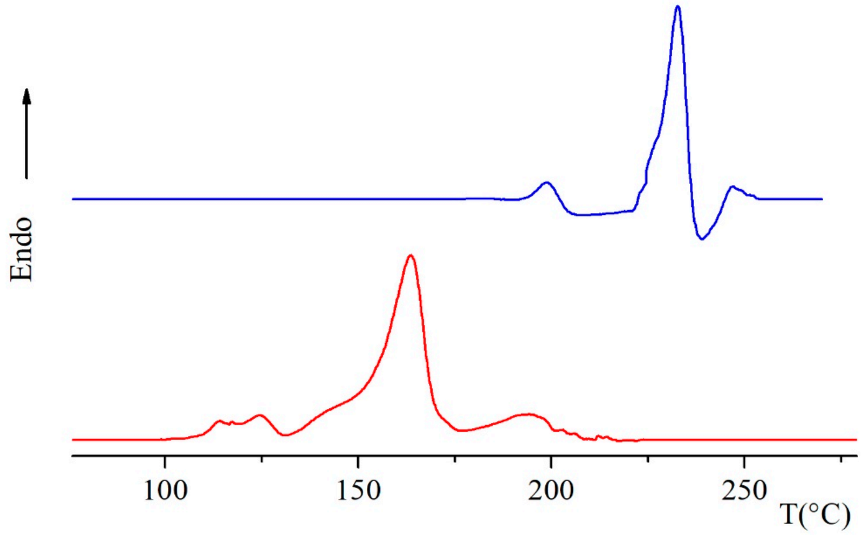

Figure S3. DSC heating curve (scanning rate of 10 °C/min, under nitrogen flow) of P2b (blue curve) and co-P2b (red curve).

© 2019 by the authors. Submitted for possible open access publication under the terms and conditions of the Creative Commons Attribution (CC BY) license (http://creativecommons.org/licenses/by/4.0/).
